# Glycoconjugates Distribution during Developing Mouse Spinal Cord Motor Organizers

**DOI:** 10.6091/ibj.1298.2015

**Published:** 2015-01

**Authors:** Elham Vojoudi, Vahid Ebrahimi, Alireza Ebrahimzadeh-Bideskan, Alireza Fazel

**Affiliations:** Dept. of Anatomy and Cell Biology, School of Medicine, Mashhad University of Medical Sciences, Mashhad, Iran

**Keywords:** Development, Glycoconjugates, Spinal cord

## Abstract

**Background:** The aim of this research was to study the distribution and changes of glycoconjugates particularly their terminal sugars by using lectin histochemistry during mouse spinal cord development. **Methods:** Formalin-fixed sections of mouse embryo (10-16 fetal days) were processed for lectin histochemical method. In this study, two groups of **horseradish peroxidase-**labeled specific lectins were used: N-acetylgalactosamine, including Dolichos biflorus, Wisteria floribunda agglutinin (WFA), Vicia villosa, Glycine max as well as focuse-binding lectins, including tetragonolobus, Ulex europaeus, and Orange peel fungus (OFA). All sections were counterstained with alcian blue (pH 2.5). **Results:** Our results showed that only WFA and OFA reacted strongly with the floor plate cells from early to late embryonic period of developing spinal cord. The strongest reactions were related to the 14, 15, and 16 days of tissue sections incubated with OFA and WFA lectins. **Conclusion:** The present study demonstrated that cellular and molecular differentiation of the spinal cord organizers is a wholly regulated process, and α-L-fucose, α-D-GalNAc, and α/β-D-GalNAc terminal sugars play a significant role during the prenatal spinal cord development.

## INTRODUCTION

During embryonic development, particularly the early period of tissue morphogenesis, certain fetal precursor cells are committed to differentiation in a complicated cellular and molecular developmental pathway [1, 2]. One of the first organizers of the nervous system is notochord that produces an important molecule namely sonic hedgehog (Shh). This molecule induces embryonic dorsal ectoderm into neuroectoderm, which forms a neural plate and finally develops into the neural tube. The neural tube has ventral (floor plat) and dorsal (roof plate) organizers for designing motor and sensory areas, respectively. The floor plate of prospective spinal cord is responsible for arrangement of all lower motor neurons in the ventral area of the spinal cord [3-5].

Developmental phenomena, such as cell differentiation and cell-to-cell interactions, in an appropriate pathway lead to the establishment of organizing centers, including floor plate of the spinal cord. In 2011, Ando's study [6] showed that if notochord is damaged on 9^th^ embryonic day, it can be replaced by the floor plate. The results from this study also demonstrated that before destruction, notochord can induce floor plate, and due to the absence of notochord, the floor plate can induce somite formation, which differentiates into sclerotome and vertebral column of spinal cord.

An investigation has also showed that the floor plate is composed of a small cellular group in the middle of anterior region of spinal cord [7]. Some studies have indicated that the floor plate precursors are similar to the cells of the notochord, while others demonstrated that this area is not composed of identical cell population [8-10]. The ventromedial cells occupy the midline of the ventral region of neural tube and produce Shh and forkhead box protein A2 [9]. Several investigations on some species such as Zebra fish, chickens, and mice have displayed that ventromedial cell heterogeneity is seen not only in mediolateral axis but also in the anterior posterior axis [8-10].

**Table 1:** Tested lectins and their main sugar specifications

** Specific lectins**	**Abbreviation**	**Carbohydrate-binding specificity**
α-L-Fuc (1,6) GlcNac	OFA	*Aleuria aurantia* (Orange peel fungus)
α-L-Fuc	LTA	*Lotus tetragonolobus* (asparagus pea)
α- L-Fuc (α1→ 2) Gal (β 1→4) GlcNac β	UEA-1	*Ulex europeus* (gorse seed)
α GalNAc (1→3) GalNAc	DBA	*Dolichos biflorus* (horse gram lectin)
α -D-GalNAc and β-D-GalNAc	WFA	*Wisteria floribunda* agglutinin
β Gal 1→ 3α GalNAc > Gal	VVA	*Vicia villosa* (hairy winter vetch)
α/β-D-GalNAc > α/β-D-Gal	SBA	*Glycine max* (soybean)

***Fuc, fucose; Gal, galactose; ***GalNAc***, N-acetylgalactosamine***

The sugar compounds on the cell surface and extracellular matrix are important as messengers for tissue differentiation. These compounds, which are called glycoconjugates, are known as mucopoly-saccharide complexes [11-13]. In similar previous studies, it has been shown that the presence of glycoconjugate-rich compounds in somato-sensory cortex of the brain is necessary during nervous system development [14, 15]. These macromolecules, which are located in the cell surface and extracellular matrix, contain different carbohydrates such as glycolipids, glycoproteins, glycophospholipids, and proteoglycans. In many developmental systems, cell surface glycoconjugates, particularly definite terminal residues (such as sialic acid, fucose, and N-acetylgalacto-samine) play a significant role in developmental events [16, 17]. These compounds also have a critical function in cell-cell and cell-extracellular matrix interactions as well as tissue differentiations. When performing their function, these glycoconjugates have two different destinations: the first destination is covered with sialic acid or some other molecules and the second one is broken by means of various enzymes [18, 19]. All of these compounds appear simultaneously with developmental events during different stages of organogenesis [19, 20].

Lectin histochemistry is one of the best experimental procedures for determining the distribution of glycoconjugates and their changes on the cell surface during embryonic development. The characteristic feature of lectins is their ability to bind the terminal carbohydrate residues. Lectins also can recognize isomeric glycan with similar compounds. Therefore, lectins are useful diagnostic agents for evaluation and follow-up of tissue differentiation in the histochemistry labs [21-23].

The aim of this study was to determine distribution and changes of typical cell surface glycoconjugates and their microenvironment on the spinal motor organizers during embryonic development. 

## MATERIALS AND METHODS

***Embryo processing and tissue preparation. ***For this study, 20 BALB/c mice (15 adult virgin females and 5 males) with an average weight of 25-30 g were obtained from the Faculty of Medicine Animal Lab, Mashhad University of Medical Sciences (Iran). Females were mated with males of the same strain (three females + one male), and zero day of gestation (E_0_) was determined by using of vaginal smear method [24-27]. At gestational stages 10 through 16 days, two pregnant mice were sacrificed by chloroform under deep anesthesia, and then the embryos were dissected from the uterus and intraembryonic membranes. All embryos (eight embryos for each pregnant mouse) were washed in normal saline and fixed in formalin solution at room temperature for 3-5 days. After dehydration of the specimens by passing through a series of successive graded ethanol, the embryos were cleared with xylene and embedded in paraffin blocks. At the end, a rotary microtome (Leitz 1512, Germany) was used for cutting 5-um transverse serial sections [28-30].

***Lectin histochemistry. ***All **horseradish peroxidase**-labled lectins were purchased from Sigma Aldrich Company, USA (Table 1). Three sections of the thoracic region of each embryonic day for each lectin were chosen randomly. The specimens were deparaffinized in xylene and hydrated by passing in a series of reduced grade ethanol. Afterward, the sections were washed in 0.1 M PBS (pH 7.4) for 10 minutes. Intrinsic peroxidase activity was blocked by hydrogen peroxide/methanol (1:100) in a dark chamber at room temperature for 45 minutes [28, 29]. The selected sections of spinal cord were placed in a solution containing 10-20 μg/ml of lectin-**horseradish peroxidase **conjugate in 0.1 M PBS (pH 7.2) for two hours. After washing with PBS, the sections were incubated in a 0.03% diaminobenzidine at room temperature for 15 minutes [28, 29]. All tissue sections were counterstained with 1% solution of Alcian blue (pH 2.5) for 5 minutes. Finally, the sections were dehydrated, cleared, and mounted on glass slides. The affinities of the tested lectins were separately observed by three blinded examiners using a light microscope (Olympus AH-2, Japan, magnification ×200). Grading was carried out according to the intensity of the lectin reactions to each specimen: from negative (-) to severe (+++) [28, 29].

**Table 2:** Summary of reactions of the tissue sections incubated with specific lectins

**Location** **of** **lectin**		**Floor** **plate** **of neural tube**									**Basal** **plate** **of neural tube**							
**Lectins **		**Late morphogenesis**				**Early ** **morphogenesis**					**Late ** **morphogenesis**				**Early ** **morphogenesis**			
**GD**		**16**	**15**	**14**		**13**	**12**	**11**	**10**		**16**	**15**	**14**		**13**	**12**	**11**	**10**
OFA		+++	+++	+++		++	++	++	+		++	++	++		+	_	_	_
LTA		_	_	_		_	_	_	_		_	_	_		_	_	_	_
UEA-1		_	_	_		_	_	_	_		_	_	_		_	_	_	_
DBA		_	_	_		_	_	_	_		_	_	_		_	_	_	_
WFA		+++	+++	+++		++	++	++	+		+	+	+		+	_	_	_
VVA		_	_	_		_	_	_	_		_	_	_		_	_	_	_
SBA		_	_	_		+	+++	++	+		_	_	_		+	++	+	_

GD, gestational day; OFA, orange peel fungus; LTA, *Lotus tetragonolobus*; UEA-1, *Ulex europeus*; DBA, *Dolichos biflorus; *WFA,* Wisteria floribunda* agglutinin; VVA,* Vicia villosa*; SBA*, Glycine max; *Negative (-), Week (+), Moderate (++), Severe (+++)

## RESULTS

**Fig. 1:**
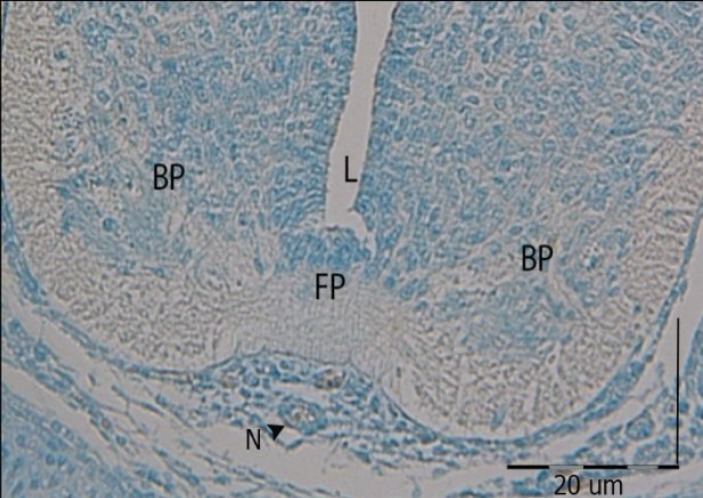
Cross section photomicrograph of GD14 of developing mouse spinal cord incubated with DBA lectin. DBA did not react with any part of the developing spinal cord. BP, basal plate; FP, floor plate; L, lumen. N, notochord

In this study, lectin histochemical technique was carried out on histological sections of mice embryos during at both early gestational day (GD10) to GD13 and late (GD14 to GD16) embryonic periods of morphogenesis. All results are shown in Table 2. The result showed that all lectins, including Vicia villosa (VVA), Lotus tetragonolobus (LTA), Dolichos biflorus (DBA), and Ulex europaeus (UEA-1) did not react with any part of developing motor organizer at morphogenic stages (Fig. 1). 

The sections obtained from the floor plate of 10^th^ day of gestation indicated weak reactions with Wisteria floribunda (WFA), Glycine max (SBA), and Orange peel fungus (OFA. However, weak reaction with SBA lectin disappeared after GD14. Also in the floor plate, moderate reactions with OFA and WFA were observed on GD14 and then increased, and the reaction was evaluated as strong during GD16. 

Positive reactions of OFA lectin were observed in all areas of spinal cord at all stages of morphogenesis, especially this reaction was severe in lateral funiculi, ependymal area, and the nerve fibers of floor plate.

**Fig. 2:**
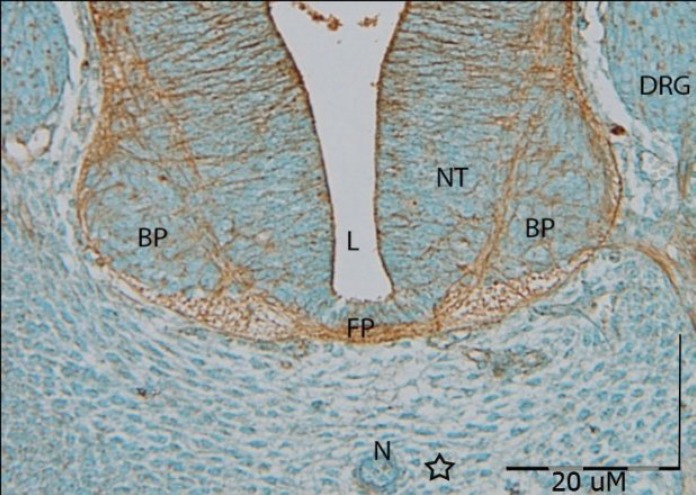
Cross section photomicrograph of GD12 of developing motor area of the neural tube incubated with OFA lectin. Cell mass around the notochord (N) can be seen ( ). It seems that in basal plate (BP) of the neural tube (NT), advanced differentiation was happened. Dorsal root ganglion (DRG) cells show a weak reaction. ***FP, floor plate; L, lumen. N, notochord***

***Reactions of glycoconjugates with tested lectins on different*** ***gestational*** ***days. ***Reactions of OFA were observed in all areas of neural tube at all stages of embryonic days. The positive reaction was severe in lateral funiculi, floor plate, and ependymal area (Fig. 2). The tissue sections of floor plate from GD10 and their surrounding fibers showed a relatively weak reaction to OFA lectin. This reaction was increased during the 11^th^ embryonic day and evaluated as severe reaction on GD14 to GD16. Beginning of positive reaction in basal plate was weak at GD11. The reaction of developing basal plate to this lectin was evaluated as weak during the 13^th^ day and then showed a relatively moderate reaction in this area and continued moderately until GD16.

**Fig. 3:**
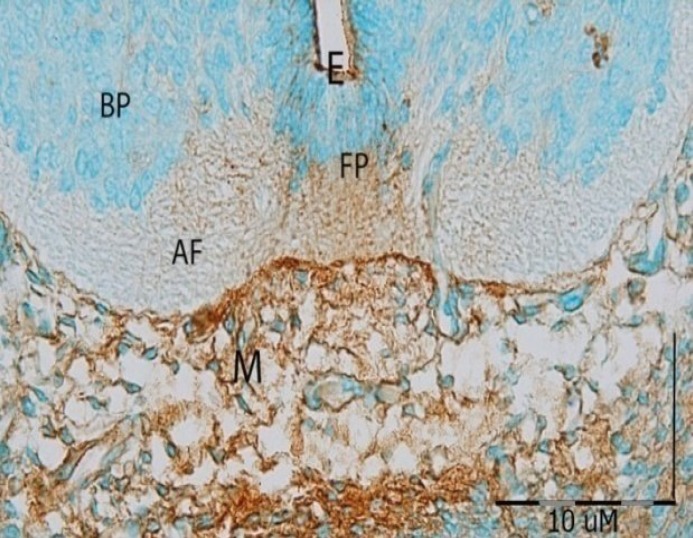
Cross section photomicrograph of GD14 of ***developing mouse*** spinal cord incubated with WFA lectin. Weak reaction is observed in the neural tube except in the floor plate (FP) and ependymal cells (E) of this area. Also, anterior funiculus (AF) showed moderate reaction with WFA. ***BP, basal plate; AF, anterior funiculus.***

**Fig. 4:**
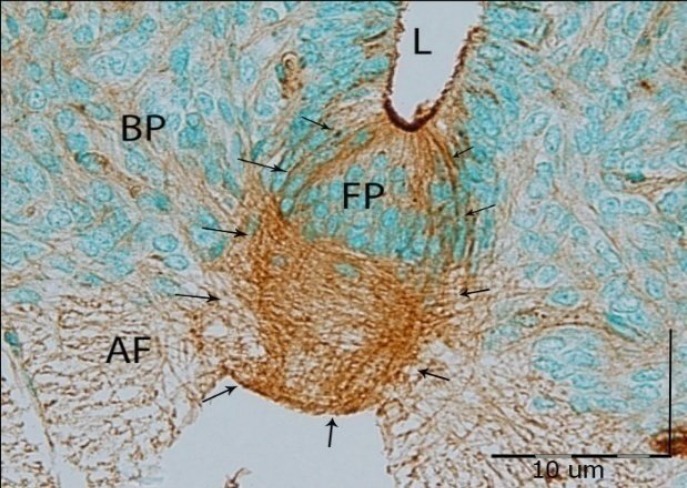
Cross section of GD16 motor zone of the developing spinal cord incubated with WFA lectin. In the ependymal zone, severe reaction around the anterior organizer (FP) is observed (Arrows). This lectin also is reacted weakly with the basal plate (BP) and anterior funiculus (AF) fibers. L, lumen.

***Wisteria floribunda*** ***agglutinin.*** Positive reaction was started both in floor plate and in roof plate, but this reaction was severe especially in floor plate fibers and anterior ependymal cells. Therefore, it can be concluded that some fibers of the anterior funiculus have commenced weak reaction during the GD13 and continued weakly until GD16. WFA had similar reaction to OFA lectin in the floor plat area; nevertheless, specific and severe reaction to this lectin was only found in the floor plate of embryonic spinal cord. Also, WFA strongly reacted with the cartilage cells near the spinal cord (Figs. 3 and 4).

Photomicrographs of tissue sections from GD10, which were incubated with SBA lectin, revealed the weak reaction of the organizer cells to spinal cord. The intensity of this reaction was increased and evaluated as moderate in anterior funiculus of 11^th^ day of early morphogenesis. Finally, the severe reaction to this lectin was related to tissue samples obtained from 12^th^ day of early morphogenesis. During another developmental day, no positive reaction was observed in neural structures containing SBA lectin. In contrast, blood vessels of GD14 were detected by this lectin, especially in the anterior funiculus.

## DISCUSSION

Neurulation or neural tube formation refers to the folding process in prenatal development, which includes the conversion of the neural plate into the neural tube. Shh signaling influences on induction of neural plate formation has been well recognized. One of the earliest visible structures in the neural tube is ventral organizer, also known as the spinal floor plate. This center, as a motor zone organizer, produces a new wave of Shh molecule. Cell fate specifications in the motor area of the neural tube (basal plate), for both neuronal and glial cells, is dependent on the presence of critical molecules within the floor plate during embryonic development. In addition to the cell differentiation, determination of an accurate pathway of developing motor neurons in spinal cord is mediated by floor plate organizer [3, 30]. Netrin and Slit are two morphogens produced by floor plate cells. Netrin acts as an attractant molecule to guide the motor axons crossing and the function of Slit is inhibiting axonal crossing of the neural tube [24-26]. 

Glycoproteins including fucose, galactose, and N-acetylgalactosamine as well as another unknown glycoconjugates on the cell surface and extracellular matrix of the motor zone are working together during spinal cord morphogenesis. Therefore, this study aimed to determine developmental timing of distribution of certain glycoconjugates within the floor plate and their motor neuron cells and neuronal process differentiation. In similar studies, by using lectin histochemistry, the efforts to characterize distribution of glycoconjugates in various tissues were performed at different stages of development [27, 31-33].

Among the lectins used in this study, OFA and WFA showed different reactions with the cells of floor plate, small parts of the developing basal plate of spinal cord and their extracellular matrix. Our findings demonstrated that the presence of fucose-containing glycoproteins (such as α-L-Fuc1) and also N-acetyl-galactosamine-containing glycoproteins (such as GalNAc) in the floor plate has not reported yet. As shown in Table 1, OFA, LTA, and UEA-1 lectins attach to the fucose terminal sugar. Similar to one previous study [34], the attachment of these lectins to terminal and pre-terminal sugars and their spatial position in the sugar chain may differ. 

In case of fucose reaction with the ventral part of developing spinal cord, it is generally believed that OFA binds preferentially to fucose by α(1→6) linkage to the GlcNac of the N-linked glycans. In addition, it binds to the terminal fucose with an α(1→3) or α(1→4) linkage. 

In the present study, LTA and UEA-1 lectins indicated no reaction with floor and roof plates, and only OFA showed a positive reaction during morpho-genesis in these areas. Thus, it could be supposed that only the recipient of GlcNac(1→6)α-L-fucose is the fucose that has a key role in development of two organizers in these areas. Furthermore, other connections between the fucose and pre-terminal sugar were not observed in these areas during morpho-genesis. The same group of lectins, including SBA, VVA, WFA, and DBA reacts with the GalNAc-containing cells; however, due to the dissimilar binding of these molecules, mentioned lectins have different interactions in different embryonic stages.

Prior studies have confirmed that there are 11 different types of GalNAc-containing glycoprotein that binds to specific lectins in the mammalian tissues [35, 36]. Based on our results, among all of the lectins, VVA and DBA (specific for GalNAc) did not show any reaction with none of the specimens of fetal period. It can be assumed that despite of the importance of GalNAc presence in floor plate development, the above mentioned lectins are not able to identify GalNAc on the cell surface of organizers. It seems that the certain glycoconjugates containing α-L-Fucose, α-D-GalNAc, and α/β-D-GalNAc sugars play an important role in development and final differentiation of spinal motor organizer cells.

The present study demonstrates that the cellular and molecular differentiation of the spinal cord organizers is a wholly regulated process, and α-L-fucose, α-D-GalNAc, and α/β-D-GalNAc terminal sugars could play a critical role during the prenatal spinal cord development.
